# A double-blinded randomized controlled trial of silymarin for the prevention of antituberculosis drug-induced liver injury

**DOI:** 10.1186/s12906-015-0861-7

**Published:** 2015-09-23

**Authors:** Chote Luangchosiri, Ammarin Thakkinstian, Sermsiri Chitphuk, Wasana Stitchantrakul, Supanna Petraksa, Abhasnee Sobhonslidsuk

**Affiliations:** Division of Gastroenterology and Hepatology, Department of Medicine, Faculty of Medicine, Ramathibodi Hospital, Mahidol University, Bangkok, Thailand; Section for Clinical Epidemiology and Biostatistics, Faculty of Medicine, Ramathibodi Hospital, Mahidol University, Bangkok, Thailand; Research Center, Faculty of Medicine, Ramathibodi Hospital, Mahidol University, Bangkok, Thailand; Division of Gastroenterology and Hepatology, Department of Medicine, Faculty of Medicine, Ramathibodi Hospital, 270 Praram 6 Road, Bangkok, 10400 Thailand

**Keywords:** Drug-induced liver injury, Hepatotoxicity, Tuberculosis, Silymarin, Antioxidant

## Abstract

**Background:**

Hepatitis is a common adverse effect of antituberculosis drugs. Silymarin prevented drug-induced hepatoxicity in animals with anti-oxidative mechanisms but its effect in human has been unknown. We aimed to evaluate the efficacy of silymarin for preventing antituberculosis-drug induced liver injury (antiTB-DILI) in patients with tuberculosis.

**Methods:**

A double-blind randomized placebo-controlled trial was performed. Tuberculosis patients were randomly allocated to receive placebo or silymarin. The outcomes of interests were antiTB-DILI and the maximum liver enzymes at week 4. Antioxidative enzymes (i.e., superoxide dismutase (SOD), glutathione and malondialdehyde assays) were assessed. The risks of antiTB-DILI between the two groups were compared. A number need to treat was estimated.

**Results:**

A total of 55 out of 70 expected numbers of patients were enrolled. There were 1/27 (3.7 %) and 9/28 (32.1 %) patients who developed antiTB-DILI in the silymarin and the placebo groups. Risk reduction was 0.28 (0.10, 0.47), i.e., receiving silymarin was 28 % at lower risk for antiTB-DILI than placebo. This led to prevention of 28 patients from being antiTB-DILI among 100 treated patients. Median (IQR) of ALT levels at week 4 in the placebo and the silymarin group were 35.0 (15, 415) IU/L and 31.5 (20, 184) IU/L (*p* = 0.455). The decline of SOD level at week 4 in the silymarin group was less than the placebo group (*p* < 0.027).

**Conclusions:**

Silymarin reduced the incidence of antiTB-DILI. The benefit of silymarin may be explained from superoxide dismutase restoration. Larger clinical trials are required to confirm the result of our small study [Clinicaltrials.Gov Identifier Nct01800487].

## Background

Tuberculosis is one of the leading causes of death among infectious diseases in developing and undeveloped countries [1]. The standard treatment regimen of antituberculosis drugs during an intensive phase consists of isoniazid, rifampicin and pyrazinamide. In many countries, ethambutol is added in the intensive regimen during the initial two months of treatment to improve the efficacy of anti-tuberculosis therapy and shorten the total duration of treatment [2].

Hepatotoxicity, a common adverse effect of anti-tuberculosis drugs, varies from asymptomatic elevation of liver enzymes to fulminant hepatic failure. Antituberculosis-drug induced liver injury (antiTB-DILI) results in increased morbidity and mortality, treatment withdrawal, drug interruption, dose reduction and selection of drug-resistant organisms [3, 4]. The incidence rate of isoniazid-related hepatotoxicity is 1.6 % when it is given alone and it increases to 2.6 % when rifampicin is added to the treatment regimen [5]. Pyrazinamide is a potential hepatotoxic drug which can cause dose-related hepatotoxicity [6]. Risks and the severity of liver injury go up when pyrazinamide, isoniazid and rifampicin are given in combination in the standard regimen [7–9]. The incidence rates of anti-TB-DILI reported in the standard treatment regimens vary from 19.9 % to 27.7 % during the first two months of treatments [10, 11].

The exact mechanism of liver injury relating to antituberculosis drugs is still unknown. Drug-induced liver injury from isoniazid and pyrazinamide may share similar mechanisms, i.e. through the pathways of escalating oxidative stress and increased oxygen free radical regeneration [12–14]. Previous studies have reported that some drugs and herbal medicine, such as garlic or N-acetylcysteine, can prevent and reduce hepatotoxicity from antituberculosis drugs [12, 15, 16].

Silymarin, a traditional herbal drug extracted from milk thistle (*Silybum marinums*) seeds, has been used as a supplement remedy for hepatoprotection [17, 18]. The main components of silymarin comprise silybin, silydianin, silychrisin and isosilybin [17, 19]. All of these are derivatives of flavonols [17, 19]. Silymarin facilitates hepatoprotection through scavenging of free radicals, thereby reducing oxidative stress, restoring the function of antioxidative enxymes and generating cell membrane stabilization [17, 18, 20, 21]. From previous studies in an experimental animal model of anti-tuberculosis related DILI, it demonstrated that silymarin has a significant hepatoprotective effect [22, 23].

However, there has never been report of the benefit of silymarin for the prevention of antiTB-DILI in human. From a randomized controlled trial of silymarin in acute hepatitis, it showed that silymarin may be effective in improving symptoms of acute hepatitis and jaundice [24]. To date, silymarin has a high safety profile which has been confirmed from a large number of studies [24]. So far, serious adverse effects of silymarin have not been reported in both animals and human. We therefore aimed to assess the efficacy of silymarin in reducing hepatotoxicity related to the treatment of a standard combined regimen of antituberculosis drugs.

## Methods

### Patients

Patients who met the following criteria were enrolled to the study: those diagnosed with pulmonary tuberculosis from positive acid-fast staining of sputum and/or typical pulmonary tuberculosis findings from chest x-ray films, aged over 18 years, planned to receive a standard anti-tuberculosis treatment regimen with isoniazid (5 mg/kg/day), rifampicin (10 mg/kg/day), pyrazinamide (25 mg/kg/day) and ethambutol (15 mg/kg/day) in the first two months, and were willing to participate with the study and gave written consent forms. Exclusion criteria included active liver diseases (chronic viral hepatitis, autoimmune hepatitis, alcoholic hepatitis, Wilson’s disease, hemochromatosis, or cirrhosis), acquired immune deficiency syndrome, concurrently taking of herbal medicine, significant alcohol consumption (more than 20 g/day), pregnant or lactating women. In addition, patients with elevated serum alanine aminotransferase enzyme (ALT) (more than 2 times of upper normal limit) prior to enrollment were excluded from the study.

### Study design

A double-blinded randomized controlled trial was performed between January 2012 and December 2012 at Faculty of Medicine Ramathibodi Hospital, Mahidol University, Bangkok, Thailand. The study protocol was approved by the Committee on Human Rights Related to Research Involving Human Subjects and it was carried out according to the Good Clinical Practice Guideline. Written informed consents were obtained before enrollment. This study followed the CONSORT guidelines for randomized controlled trial and the study protocol was registered at www.clinicaltrials.gov (NCT01800487).

### Randomization and blinding methods

A computerized-based randomization with a block of two and four was performed by a statistician who was not involved in recruitment of patients. The randomization list was then sealed in an opaque envelope. Once patients met the inclusion criteria, a research assistant opened envelops and treatments were assigned to patients accordingly. Patients, research assistants, and doctors did not know the details of the study drugs that the patients received.

### Treatment protocol

Patients with newly-diagnosed pulmonary tuberculosis were referred from pulmonary and infectious clinics. Anti-tuberculosis drugs were started with the dosages that were calculated according to patient’s body weight. Subjects were randomly assigned to receive either silymarin or placebo (with similar appearance with the study drug) in concealed allocation manners on the first day of antituberculosis treatment.

The protocol was carried out in a double-blinded fashion. One tablet of silymarin (140 mg) or placebo was taken three times a day along with antituberculosis drugs. Study subjects were emphasized to make records when taking anti-tuberculosis and the study drugs. The remaining pills were counted on the days of follow-up to check patient compliance and adherence. Adverse effects were recorded. Alcohol, herbal, and over-the-counter drugs were prohibited throughout the study period. Patients were followed up for clinical and liver enzyme assessment at week 2 and 4 after the beginning of the study.

### Outcome of interests

The efficacy of silymarin was compared with placebo for two purposes. The primary outcome of the study was to determine the maximum ALT level within 4 weeks after treatment. (Timing of week 4 was chosen because, from our observation in a pilot study, AST and ALT started to rise at week 4, not week 8. Moreover, it was not ethical to continue the study to week 8 or after if the rising of liver enzymes already occurred at week 4) The secondary outcome of interest was the development of antiTB-DILI, which was defined as presence of the following criteria: 1) having at least one of the following events: an elevation of serum ALT level more than 2 times above upper normal limit, a rise in serum total bilirubin level to more than 1.5 mg/dl, or any increase in ALT level above baseline levels combined with anorexia, nausea, vomiting, or jaundice, 2) no other explainable causes of elevation of liver enzymes, and 3) normalization of liver enzymes after withdrawal of antituberculosis drugs [25, 26]. Severity of antiTB-DILI was classified based on World Health Organization (WHO) guideline as follow: grade 0 for ALT level <1.25 times normal; grade 1 for ALT level 1.25 to 2.5 times normal; grade 2 for ALT level 2.6 to 5.0 times normal; grade 3 for ALT level 5.1 to 10.0 times normal; grade 4 for ALT level >10 times normal, or ALT >250 IU/L if accompanied by symptoms (e.g., nausea, vomiting, abdominal pain, jaundice) [27, 28]. The treatment regimen was terminated after patients developed antiTB-DILI, whereas the antituberculosis regimen was modified according to the patient condition.

In addition, antioxidative enzymes (AOE) were also assessed at baseline and at week 4 after the initiation of antituberculosis drugs, including superoxide dismutase (SOD) activity, glutathione level and malondialdehyde (MDA) activity. SOD activity in plasma was estimated by using Superoxide Dismutase activity assay Kit (BioVision, CA USA). SOD Activity Assay Standard Curve between percent inhibition and SOD standard units was used to determine SOD activity. Glutathione was measured by fluorometric method using Glutathione assay Kit (BioVision, CA USA). Malondialdehyde (MDA), a by-product representing the level of lipid peroxidation, was measured by using the Lipid peroxidation (MDA) Assay Kit (BioVision, CA USA. The reactions of SOD, glutathione level and MDA activity were measured by Infinite® 200 PRO microplate reader (Tecan, Switzerland).

Finally, adverse events (i.e., decreased appetite, fatigue, confusion etc.) were reviewed from direct questioning and self-recording on the follow-up days.

### Statistical analysis

Sample size was calculated based on comparing mean serum ALT between silymarin and placebo. The previous study reported mean serum ALT after receiving anti-TB drugs treatment of 463 (SD = 69) IU/L [11]. We assumed that silymarin works well if it should be able to decrease serum ALT level at least 10 %. A total of 70 subjects (35 for each treatment group) were required to detect the difference, given type I error of 5 % and power of test of 80 %. Taking into account for the loss of follow up 10 %, a total of 80 subjects were therefore required.

Data were analyzed based on intention to treat analysis approach. Baseline characteristics were described using mean or median where appropriate for continuous data and frequency for categorical data. Student’s *t*-test was used to compare mean between treatment groups if data were normally distributed, otherwise Wilcoxon sum rank test or a quantile regression was applied to compare medians between groups. A risk ratio (RR) of having antiTB-DILI between treatment groups along with its 95 % confidence interval (CI) was estimated. A risk difference and a number needed to treat were also estimated. All analyses were performed using STATA version 13.1. *P-*value of less than 0.05 was considered statistical significance.

## Results

**Fig. 1:**
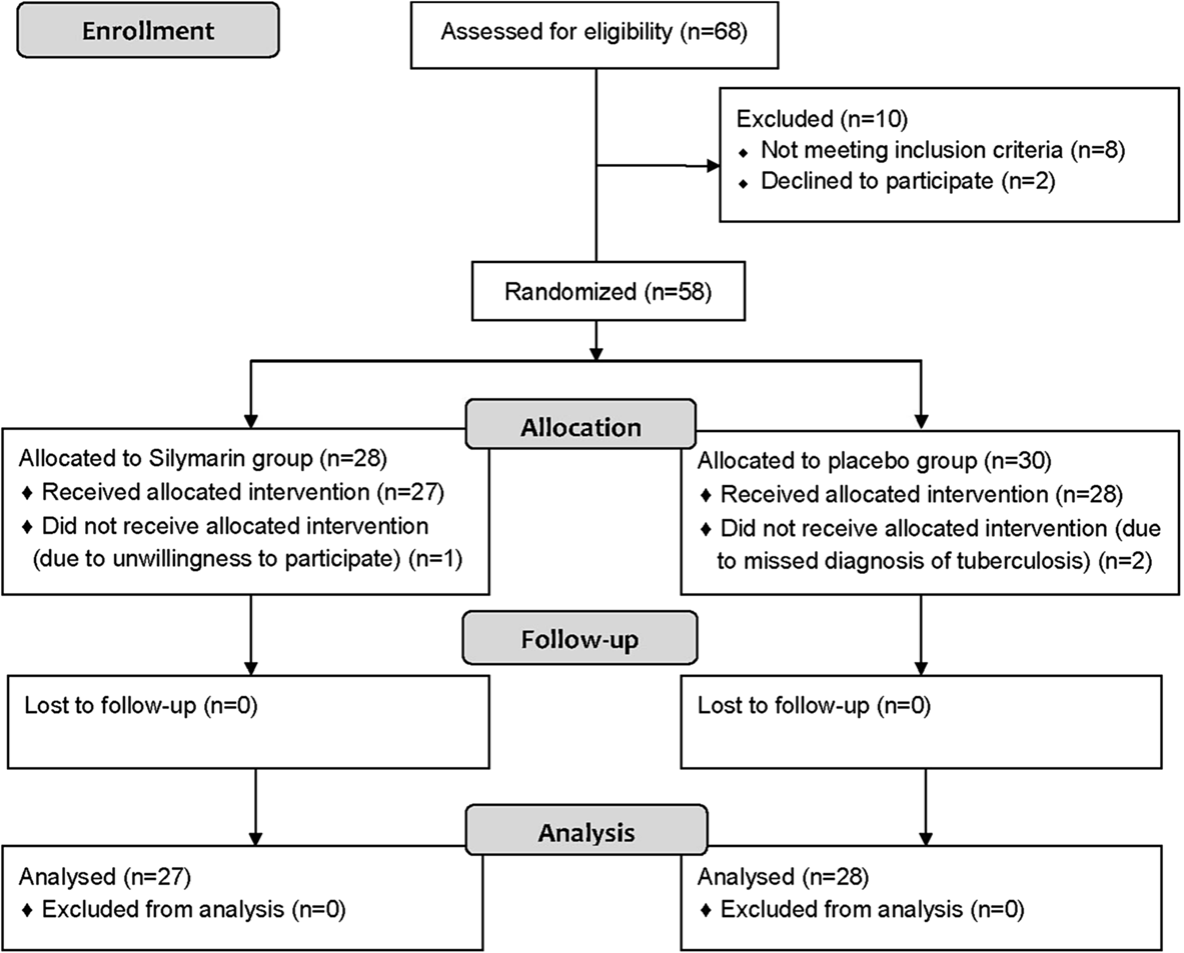
Protocol flow chart. New patients with pulmonary tuberculosis were assessed for protocol eligibility

Due to slow accrual, the recruitment was terminated prematurely after 68 patients were screened. Ten patients were excluded due to not meeting inclusion and exclusion criteria (8 patients) and decline to participate (2 patients). Fifty-eight patients were enrolled to the study (Fig. 1), 3 patients (2 in the placebo and 1 in the silymarin group) were excluded after randomization due to unwillingness to participate in 1 patient and missed diagnosis for tuberculosis in 2 patients. Of 55 patients, 27 and 28 patients were randomly assigned to receive silymarin or placebo, respectively. The trial was terminated and enrollment was stopped due to the limitation of the study duration and safety issues. Baseline characteristics were not statistically significant different between groups except for direct bilirubin, which was a little higher in the placebo than in the silymarin groups (Table 1).

**Table 1:** Baseline characteristics of patients in placebo and silymarin groups

	Silymarin	Placebo	*p*-value
	(*N* = 27)	(*N* = 28)	
Male (n, %)	10 (37.0)	12 (42.9)	0.785
Age (years)	56.0 (15, 78)	51.5 (21, 83)	0.721
BMI (kg/m^2^)	21.1 (14.1, 25.4)	21.0 (15.8, 27.0)	0.773
Dose of Isoniazid (mg/kg)	5.5 (4.0, 9.0)	5.7 (4.5, 7.7)	0.968
Dose of rifampicin (mg/kg)	10.4 (8.0, 14.0)	10.3 (9.0, 14.0)	0.965
Dose of pyrazinamide (mg/kg)	23.1 (18.6, 29.1)	23.8 (17.8, 58.0)	0.282
Dose of ethambutol (mg/kg)	18.5 (13.3, 24.9)	18.5 (12.7, 27.8)	0.859
*Liver function tests*			
ALT (IU/L)	29 (15, 66)	31 (11, 86)	0.639
AST (IU/L)	20 (13, 106)	25.5 (14, 56)	0.087
ALP (IU/L)	85 (37, 449)	89.5 (55, 508)	0.736
GGT (IU/L)	42 (17, 430)	52.5 (14, 510)	0.443
TP (g/L)	78.1 (50.5, 91.5)	76.0 (56.1, 99.4)	0.452
Alb (g/L)	35.7 (23.0, 42.0)	33.0 (16.7, 43.8)	0.639
TB (mg/dl)	0.5 (0.3, 1.9)	0.6 (0.2, 1.4)	0.285
DB (mg/dl)	0.2 (0.1, 0.7)	0.3 (0.1, 5.0)	0.015

BMI, body mass index; ALT, alanine aminotransferase; AST, aspartate aminotransferase; ALP, alkaline phosphatase; GGT, gamma-glutamyl transpeptidase; TP, total protein; Alb, albumin; TB, total bilirubin; DB, direct bilirubin

Categorical variables reported as N (%). Continuous variables reported as median (interquartile range)

Liver enzymes at week 4 after the initiation of treatment are shown in Table 2. Median (interquartile range, IQR) of ALT levels in the silymarin and placebo groups were 32 (20, 184) and 35 (15, 415) IU/L, which were not significantly different (*p* = 0.455). The incidences of antiTB-DILI at week 4 were 3.7 % and 32.1 % in the silymarin and placebo groups (Table 3). Patients who received silymarin were approximately 28 % (95 % CI: 10 %, 47 %) at significantly lower risk of having antiTB-DILI than placebo. An estimated number need to treat (NNT) was 3.5 (95 % CI: 2.11, 2.37), which could be interpreted that one patient can be prevent from antiTB-DILI in every 4 patients who have been treated with silymarin. Similarly, according to the WHO criteria for grading of DILI, the number of the patients who developed antiTB-DILI was significantly lower in the silymarin group than in the placebo group [2 (7.4 %) *vs*. 9 (32.1 %), *p* = 0.039]. Two patients in the silymarin and seven patients in the placebo groups developed WHO grade I-II DILI. Two patients in the placebo group developed WHO grade III DILI. None of the patients developed WHO grade IV DILI.

**Table 2:** Comparison of liver function tests at week 4 after initiation of antituberculosis drugs

	Silymarin	Placebo	*p*-value
	(*N* = 27)	(*N* = 28)	
ALT (IU/L)	32 (20, 184)	35 (15, 415)	0.455
AST (IU/L)	26 (14, 219)	29 (13, 386)	0.888
ALP (IU/L)	78 (37, 409)	85 (56, 249)	0.622
GGT (IU/L)	61 (13, 224)	99 (21, 366)	0.215
TP (g/L)	74.2 (25.1, 90.8)	73.3 (48.7, 93.7)	0.920
Alb (g/L)	33.6 (8, 41)	35.0 (19, 69)	0.565
TB (mg/dl)	0.6 (0.2, 2.2)	0.6 (0.2, 6.5)	0.999
DB (mg/dl)	0.3 (0.1, 1.4)	0.3 (0.1, 5.0)	0.999

ALT, alanine aminotransferase; AST, aspartate aminotransferase; ALP, alkaline phosphatase; GGT, gamma-glutamyl transpeptidase; TP, total protein; Alb, albumin; TB, total bilirubin; DB, direct bilirubin

Continuous variables reported as median (interquartile range)

**Table 3:** Comparison of risk of antituberculosis-drug induced liver injury (antiTB-DILI) between two treatment groups: Intention-to-treat approach

	Placebo	Silymarin	*p*-value	RR	RD	NNT
	(*N* = 28)	(*N* = 27)		(95 % CI)		
DILI	9 (32.1)	1 (3.7)	0.012	0.12 (0.02, 0.85)	0.28 (0.10, 0.47)	3.50 (2.11, 2.37)
Non-DILI	19 (67.9)	26 (96.3)		1		

DILI, drug induced liver injury; RR, risk ratio; RD, risk difference; NNT, number need to treat

Categorical variables reported as N (%)

The changing of antioxidative enzymes levels (**Δ**AOE) after 4 weeks of treatment (AOE at week 4 – AOE at baseline) was described in Table 4. Although the median change of serum transaminase, glutathione and MDA were not significantly different between the two treatment groups, the decline of mean SOD level at week 4 in the silymarin group was less than in the placebo group (*p* =0.027). Serious adverse effects relate to antituberculosis drugs and silymarin were not seen in this study. The incidences of mild adverse effects (nausea and dizziness) were not significantly different between the two groups (Table 5).

**Table 4:** The changing of antioxidative enzymes levels (ΔAOE) after 4 weeks of treatment (AOE at week 4 - AOE at baseline)

	ΔAOE after 4 weeks of treatment		
	Silymarin	Placebo	*p*-value
SOD^a^ (%)	−0.20 (−4.0, 6.4)	−4.41 (−6.0, 1.8)	0.027
Glutathione^a^ (ng/ul)	−0.09 (−0.3, 0.0)	−011 (−0.2, 0.0)	0.83
MDA^a^ (nmol/L)	−0.14 (−13.5, 17.1)	−9.74 (−23.8, 6.0)	0.22

AOE, Antioxidative enzymes; SOD, superoxide; MDA, malondialdehyde

^a^ΔAOE reported as median (interquartile range)

**Table 5:** Adverse events

	Placebo	Silymarin
	(*N* = 28)	(*N* = 27)
Major adverse events	0	0
Minor adverse events	3 (10.7)	3 (10.7)
• Nausea / vomiting	1	3
• Dizziness	2	0
Total	3 (10.7)	3 (10.7)

Categorical variables reported as N (%)

## Discussion

Antituberculosis drug-related hepatotoxicity is an important adverse effect that can occur during the first two months when three or four antituberculosis drugs are required to take together. The incidence rate of antiTB-DILI could reach 27.7 % during the intensive phase of therapy [11]. However, some drugs might be beneficial in preventing antituberculosis drug-related hepatotoxicity. In a recent study of animal models, silymarin was found to prevent hepatitis from antituberculosis drugs [22]. A recent randomized controlled study of silymarin could not demonstrate the effect of silymarin in the prevention of antiTB-DILI [29]. However, the study was an open-label trial, and vitamin C, which is a non-enzymatic low molecular weight antioxidant [19], was used in the control arm [29]. We conducted a double-blinded randomized controlled trial which aimed to assess the efficacy of silymarin in the prevention of antiTB-DILI. Although we could not recruit tuberculosis patients to the expected number from sample size calculation due to the limitation of study period and safety reasons, the study result showed a benefit of silymarin. The risk of antiTB-DILI in the patients who were treated with silymarin was approximately 28 % lower than placebo, i.e., 28 patients will be prevented from antiTB-DILI among 100 treated patients. However, the small sample size of subjects in both groups was a considerable limitation of our study.

**Fig. 2:**
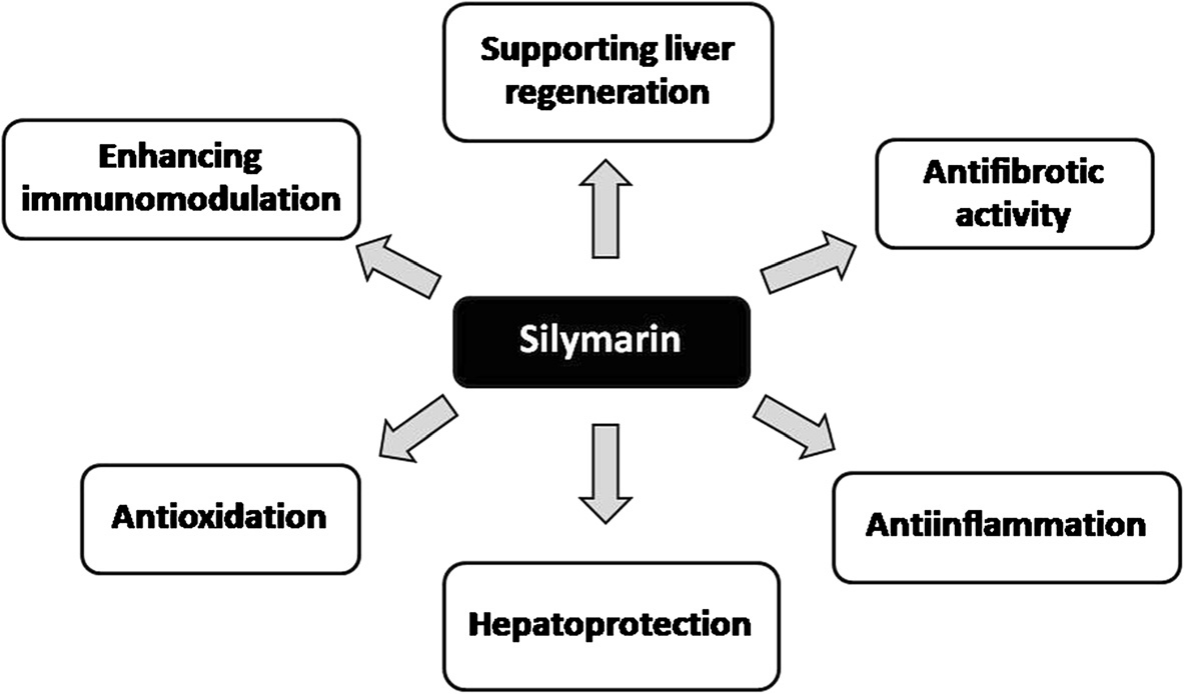
Mechanisms of action of silymarin. The positive effects of silymarin on the liver are originated from hepatoprotection, antioxidation, antiinflammation, antifibrotic activity, the promotiion of liver regeneration and immunomodulation [17, 18, 31]

The efficacy of silymarin was assessed by comparing ALT levels and the incidence of antiTB-DILI between the placebo and the silymarin groups. Because most of antiTB-DILI in this study started to appear in the first 4 weeks, not 8 weeks as described in previous reports [30], the liver enzymes at week 4 were selected for the endpoint analysis in this study. There was no patient who developed antiTB-DILI after week 4 of treatment. Although the maximum ALT level during 4 weeks after initiation of antituberculosis treatments were not different between the treatment groups [35 (15–415) vs. 31.5 (20–184) IU/L], the risk of antiTB-DILI was significantly higher in the placebo than in the silymarin group, according to the study protocol and the WHO criteria. The result of this study suggested that silymarin (140 mg) three times a day had its efficacy in the prevention of antiTB-DILI. Drug-induced hepatotoxicity leads to oxidative stress, lipid peroxidation, the reduction of phospholipids and protein synthesis as well as glutathione in the liver [23]. The mechanisms of action of silymarin and silybinin encompass hepatoprotection, antioxidation, antiinflammation, antifibrotic activity, stimulation of protein synthesis and liver regeneration, and enhancing immuno-modulatory effect as summarized in Fig. 2 [17, 18, 31]. Silymarin inhibits proinflammatory cytokines, acts as oxygen free radical scavenger and potentiates antioxidant capacity of the liver [17, 18, 23, 31]. From the results of this study, the mechanism by which silymarin is beneficial in the prevention of antiTB-DILI may be explained from superoxide dismutase restoration. An increase in the nuclear translocation of nuclear factor erythroid 2-related factor 2 (Nrf2) and decreased tumor necrosis factor (TNF)-α mRNA expression in the liver may be a protective effect of silymarin in drug-induced liver injury [32, 33].

Interestingly enough, although the patients in this study did not have the risk profiles of antiTB-DILI such as old age or malnutrition [34], we found a higher incidence of antiTB-DILI (32.1 %) than previously reported (19.9 - 22.2 %) [34]. This phenomenon may be explained from the modified criteria for the diagnosis of antiTB-DILI that was used in this study which lowered ALT levels to only 2 times above upper normal limit. When the diagnosis of antiTB-DILI was made, antituberculosis drugs and/or their dosages were modified. Patient conditions and liver enzymes were followed up until liver enzymes returned to their baseline levels. No patients developed fulminant hepatic failure or died in this study. In the silymarin group, there were mild adverse effects similar to the placebo group. There was no serious adverse effect occurring in the study. Silymarin has a safety profile that can be used in liver diseases. Only minor adverse effects such as diarrhea, nausea, vomiting and lightheadedness were reported and required only supportive treatment in some patients.

## Conclusions

From this study, it is suggested that silymarin has an efficacy to prevent liver injury from a standard combined antituberculosis drugs without significant adverse effects. Superoxide dismutase restoration may be one of the mechanisms that can explain the benefit of silymarin in the prevention of antiTB-DILI. However, larger and better designed clinical trials are required to confirm the results of our small and single-center study before silymarin can be safely recommended to prevent liver injury from antituberculosis drugs.

## Abbreviations

AntiTB: Antituberculosis
DILI: Drug induced liver injury
ALT: Alanine aminotransferase
WHO: World Health Organization
AOE: Antioxidative enzymes
SOD: Superoxide dismutase
MDA: Malondialdehyde
RR: Risk ratio
CI: Confidence interval
NNT: Number need to treat
